# Relationships between Controlling Interpersonal Coaching Style, Basic Psychological Need Thwarting, and Burnout, in Adolescent Soccer Players

**DOI:** 10.3390/ijerph17134909

**Published:** 2020-07-07

**Authors:** Verónica Morales-Sánchez, Miriam Crespillo-Jurado, David Jiménez-López, Juan P. Morillo-Baro, Antonio Hernández-Mendo, Rafael E. Reigal

**Affiliations:** Faculty of Psychology, University of Malaga, Teatinos Campus, 29071 Malaga, Spain; vomorales@uma.es (V.M.-S.); miriamcj@uma.es (M.C.-J.); davidjimenezlopez.93@gmail.com (D.J.-L.); juanpablo.morillo@gmail.com (J.P.M.-B.); mendo@uma.es (A.H.-M.)

**Keywords:** controlling coaching style, basic psychological needs, burnout, soccer

## Abstract

The aim of this paper is to analyze the relationships between a controlling interpersonal style, psychological need thwarting and burnout in adolescent soccer players and to test a structural equation model to analyze whether (a) a controlling interpersonal style is a predictor of psychological need thwarting and whether (b) psychological need thwarting is a predictor of burnout. A total of 103 male soccer players between the ages of 12 and 17 participated in the research (*M* *=* 14.91; *SD* *=* 5.56). The Controlling Coach Behaviors Scale, the Psychological Need Thwarting Scale, and the Athlete Burnout Questionnaire were used to evaluate the variables under study. The analyses revealed significant relationships between a controlling interpersonal style, psychological need thwarting and burnout. Furthermore, the proposed structural equations model, using the partial least squares (PLS) method, showed that a controlling style is a positive predictor of basic psychological need thwarting and that the latter is a predictor of burnout, as well as revealing an indirect relationship between a controlling style and burnout. This indirect effect of the controlling style variable on burnout can be enhanced (or attenuated) by the basic psychological need thwarting variable, which acts as a modulator.

## 1. Introduction

Social context has a very important impact on the development of athletes’ motivation and well-being [1,2,3]. Agents such as parents or coaches can significantly influence the sport experience, turning it into something positive or negative. It is therefore appropriate to monitor external variables that could determine the way in which sport is experienced [4,5,6,7]. Indeed, factors such as putting excessive pressure on athletes to achieve good results or failing to foster the enjoyment of physical activity can even lead them to abandon sports practice [8]. Therefore, analyzing the behavior of coaches and their style of interaction with athletes is a very interesting area of research.

Among the theoretical frameworks available to evaluate this issue, one of the most currently influential approaches that has been used to study motivation in sport is Self-Determination Theory [9,10]. This is a macro theory that analyzes the degree to which human behavior is autonomous or volitional and is composed of six mini-theories. One of these is Basic Psychological Needs Theory, which has been analyzed in detail in a number of studies. It refers to the relationships between the satisfaction or thwarting of basic psychological needs (autonomy, relatedness, and competence) and well-being or ill-being [10,11]. The thwarting of these three basic needs is considered as a stronger and more threatening experience than the absence of their satisfaction; it is the perception that the satisfaction of needs is being obstructed or actively frustrated [12]. Autonomy refers to the experience of will and disposition. When it is satisfied, people feel a sense of integrity; when they perceive it as being obstructed, they experience a sense of pressure. Relatedness denotes the experience of bonding and caring and is satisfied by connecting with and feeling meaningful to others. The undermining of relatedness entails a feeling of social alienation and loneliness. Competence refers to the experience of effectiveness and mastery. It is satisfied through opportunities to use and expand skills and experience. The thwarting of competence is experienced as a sense of ineffectiveness or even failure [13].

Studies have shown that the satisfaction of basic psychological needs contributes to an increase in intrinsic motivation, greater enjoyment of the physical activity by athletes and a higher degree of adherence to the activity [14,15]. It increases their perception of their ability and their confidence, making them feel better about the task. In addition, people who feel free to choose an activity and less obliged tend to feel more comfortable with it. Finally, an appropriate social environment and the establishment of positive interpersonal ties promote adherence [13,14,15]. It is therefore of interest to analyze how a coach’s interaction style is related to the satisfaction or non-satisfaction of basic psychological needs.

Various studies have indicated that coach behavior is decisive for the satisfaction of basic psychological needs [16,17,18]. According to Self-Determination Theory, coaches can adopt two ways of interacting with athletes: a controlling style or an autonomy-supportive style. When coaches use a controlling style, they are more authoritarian, allow less choice, put more pressure on their athletes, try to impose their way of thinking and do not attempt to be empathetic toward the athlete [19]. These behaviors are very significant, since they could have consequences for how athletes relate to the sport and how they might act in relation to it in the future.

Therefore, when coaches exhibit a controlling style of interaction, it can have negative effects on athletes, as several studies have shown, giving rise to basic psychological need thwarting, physical and psychological distress and a less satisfactory sports experience [5,17]. However, when coaches are autonomy-supportive, they promote the satisfaction of basic psychological needs and greater well-being in athletes, being more flexible and less coercive, giving more choice and tending to put themselves in the athlete’s place [20,21,22]. This leads to a more positive experience of physical activity and greater satisfaction with the task at hand [23,24].

These processes can have more or less serious consequences for the athlete. Among the most negative, one of the constructs related to ill-being in sport that has attracted the most interest from researchers is burnout [25,26]. According to Raedeke [27], burnout is a syndrome caused by physical and emotional exhaustion, a low sense of achievement and sport devaluation. Burnout in the field of physical activity and sport is considered a state that may be associated with negative variables such as the abandonment of physical practice, decreased physical performance, poor enjoyment of the sport activity practiced or increased injuries [28]. Athletes with high levels of burnout often develop behaviors that distance them from sport, explicitly manifesting a separation from physical practice [29]. Various researchers have observed that, in situations of sports stress, athletes with greater mental toughness have a greater ability to protect themselves against its adverse effects, developing fewer health problems and less burnout [30].

Several studies have related the motivational climates generated by coaches to burnout in athletes [31,32,33,34]. Castillo et al. [32] found that a controlling coaching style was positively related to basic psychological need thwarting, and this in turn was related to burnout, in under-14 category soccer players. Barbosa-Luna et al. [33] observed that a perception of tasks involving motivational climates was positively related to self-determined motivation and this in turn was negatively related to burnout in university athletes. Similarly, Mars et al. [5] analyzed a sample of under-14 and under-16 category soccer players, finding a positive correlation between a controlling coaching style, basic psychological need thwarting and burnout.

In view of their importance in the processes of sports adherence and player well-being, the aim of this study was to explore the relationships between a controlling interpersonal style, psychological need thwarting and burnout in adolescent male soccer players. In addition, a structural equation model was tested to determine whether (a) a controlling interpersonal style is a predictor of psychological need thwarting and whether (b) psychological need thwarting is a predictor of burnout. This model has previously been tested in a study with an under-14 sample [32] and later in another study with an under-16 sample [5], which was an extension of the previous study. In the present investigation, the age range has been extended to under-18, as we wished to continue exploring this model in a group of older soccer players. Furthermore, in this study, generalizability analyses are proposed and the structural equations model has been applied using the partial least squares (PLS) method, which lends consistency to the observed results.

## 2. Materials and Methods

### 2.1. Participants

A total of 103 male soccer players participated in the study. They belonged to the Alhaurín de la Torre C.F club (Malaga, Spain). All the participants were federated soccer players and competed in local and regional leagues. Their ages were from 12 to 17 years (*M* = 14.91; *SD* = 5.56). All of them carried out three training sessions per week with a duration of 90 minutes per session and had a minimum of four years’ experience of sports practice. The sampling was not probabilistic; it was chosen for convenience. Participants had spent at least two years on the same team and at least one year with the same coach.

### 2.2. Instruments and Measures

Perception of the coach’s controlling interpersonal style was evaluated with the Spanish version [35] of the Controlling Coach Behaviors Scale [20]. This questionnaire consists of 15 items and four dimensions: controlled use of rewards, negative conditional support, intimidation and excessive personal control. Each item began with the phrase “on my soccer team”, and the answers were recorded on a seven-point Likert scale ranging from 1 (strongly disagree) to 7 (strongly agree). In previous research, the Spanish version has shown adequate validity and a reliability between 0.66 and 0.83 (Cronbach’s Alpha) [20,35]. For this study, the internal consistency values (Cronbach’s Alpha) were controlled use of rewards at 0.74, negative conditional support at 0.83, intimidation at 0.77 and excessive personal control at 0.71. Average scores were calculated for each factor; a higher value meant a more controlling style.

Basic psychological need thwarting was analyzed using the Spanish version [36] of the Psychological Need Thwarting Scale [12]. This scale has 12 items grouped in three dimensions: competence, autonomy and relatedness. Each item began with the phrase “on my soccer team” and the answers were recorded on a seven-point Likert scale ranging from 1 (strongly disagree) to 7 (strongly agree). In previous research, the Spanish version has shown a reliability of 0.86 in the total scale and alphas of the three subscales ranging from 0.70 to 0.75 [12,37]. For this study, the internal consistency values (Cronbach’s Alpha) were competence at 0.75, autonomy at 0.71 and relatedness at 0.73. Average scores were calculated for each factor; a higher value meant greater psychological need thwarting.

Burnout was evaluated using the Spanish version [36] of the Athlete Burnout Questionnaire [38]. This scale has 15 items grouped in three dimensions: physical/emotional exhaustion, sport devaluation and reduced sense of accomplishment. The answers were recorded on a five-point Likert scale ranging from 1 (almost never) to 5 (almost always). In previous research, the total scale in the Spanish version has shown an alpha of 0.87 [36]. For this study, the internal consistency values (Cronbach’s Alpha) were physical/emotional exhaustion at 0.82, reduced sense of achievement at 0.75 and devaluation of sports practice at 0.77. Average scores were calculated for each factor; a higher value meant greater burnout.

### 2.3. Procedure

First, the management of the sports club was contacted and asked to collaborate in carrying out the research. Next, the study objective was explained to the players, relevant queries were resolved and the players were told that participation would be voluntary. Their consent was obtained; as they were minors, it had to be signed by their parents/legal guardians. The players completed the instruments in the final stretch of the 2016–2017 season, during the month of May, so that all the players in the study would have worked for at least one season with their coach. The data collection was done before a training session, in a suitably equipped room, always under the supervision of an investigator to resolve possible queries. The instruments were self-administered. Throughout the research process, the ethical principles of the Declaration of Helsinki [39] were followed and the study was approved by an ethics committee at the University of Malaga.

### 2.4. Data Analysis

**Figure 1:**
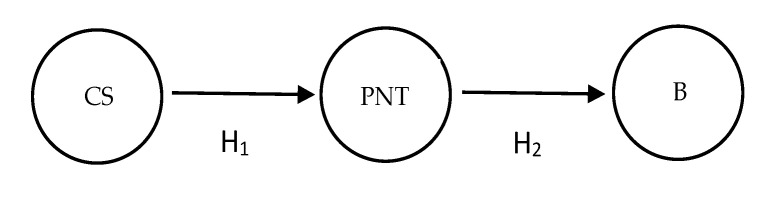
Study hypotheses. Note: CS: controlling style; PNT: psychological need thwarting; B: burnout; H_1_: hypothesis 1; H_2_: hypothesis 2.

The data were subjected to descriptive and inferential analysis. First, a generalizability analysis was performed to find out whether the results obtained could be generalized. Second, the descriptive statistics and the Kolmogorov–Smirnov test were calculated. Third, Pearson’s bivariate coefficient was used to analyze the correlations between the measures under study. Fourth, a structural equations model was tested using the partial least squares (PLS) methodology, which works with blocks of variables and estimates the parameters of the model by maximizing the explained variance of all the dependent variables [40]. The significance of the parameters was established through a bootstrap resampling procedure of 5000 samples. The hypotheses raised by this model are (1) that a controlling style will act as a positive predictor of psychological need thwarting and (2) that the psychological need thwarting of the players will act as a positive predictor of burnout (Figure 1). SPSS software version 20.0 and SmartPLS 3 (v.3.2.8) [41] were used for the statistical processing of the data.

## 3. Results

### 3.1. Generalizability Analysis

In order to determine whether the results obtained could be generalized, generalizability analyses were performed (Table 1). For this purpose, a cross-faceted design on the (q) (f)/(p) model was used, where (q) = questionnaire, (f) = factor, and (p) = participants. This model was based on a general structure with all the questionnaires, and generalizability coefficients greater than 0.94 were obtained (relative G = 0.95 and absolute G = 0.94). In addition, different analyses were performed for each questionnaire. For the Controlling Coach Behaviors Scale, a generalizability analysis was performed with a cross-faceted design on the (f)/(p) model, where (f) = factor and (p) = participants, and generalizability coefficients greater than 0.78 were obtained (relative G = 0.81 and absolute G = 0.78). The same model, (f)/(p), was used for the Psychological Need Thwarting Scale and generalizability coefficients greater than 0.80 were obtained (relative G = 0.88 and absolute G = 0.80). Likewise, the same model, (f)/(p), was used for the Athlete Burnout Questionnaire and generalizability coefficients greater than 0.97 were obtained (relative G = 0.99 and absolute G = 0.97). These data confirm the generalizability of the numerical structure of the study sample.

### 3.2. Descriptive Statistics and Correlation Analysis

Firstly, the scores obtained by the study participants are presented. Table 2 shows the descriptive statistics of the study variables. As can be seen, the scores obtained indicate that the data followed a normal distribution for the set of variables analyzed.

Secondly, correlation analyses were performed to determine the associations between the study variables. The correlation analyses carried out (Table 3) indicate the existence of relationships between the study variables, with the most significant being those established between devaluation of sports practice and physical/emotional exhaustion, reduced sense of accomplishment and autonomy thwarting, as well as those established between competence thwarting and reduced sense of accomplishment and between competence thwarting and autonomy thwarting.

### 3.3. Structural Equation Analysis

**Figure 2:**
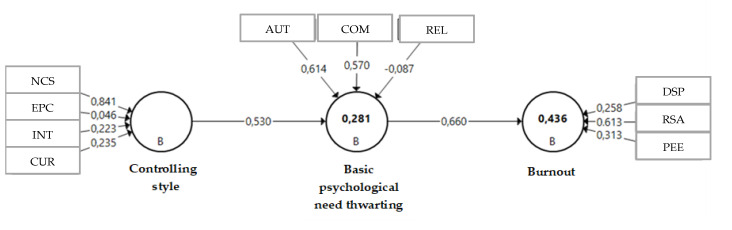
Standardized model solution. Note. NCS: negative conditional support; EPC: excessive personal control; INT: intimidation; CUR: controlled use of rewards; AUT: autonomy; COM: competence; REL: relatedness; RSA: reduced sense of accomplishment; DSP: devaluation of sports practice; PEE: physical/emotional exhaustion.

Thirdly, having observed the existence of relationships between the variables, we proposed a structural equation model to examine the study hypotheses: (a) a controlling interpersonal style is a predictor of psychological need thwarting and (b) psychological need thwarting is a predictor of burnout. These variables are made up of various indicators. The controlling style variable comprises four factors: negative conditional support, excessive personal control, intimidation and controlled use of rewards. The basic psychological needs thwarting variable is composed of three indicators: autonomy, competence and relatedness need thwarting. Finally, the burnout variable is also made up of three indicators: devaluation of sports practice, reduced sense of accomplishment and physical and emotional exhaustion. Figure 2 shows the standardized solution of the proposed model. Following Cepeda-Carrión et al. [42] and Henseler, Hubona, and Ray [43], the first aspect to evaluate within the model is the overall goodness-of-fit in both the estimated and the saturated model.

Table 4 shows the different adjustment indexes of the two models, as the standardized root mean square residual (SRMR) value of the measurement model must be less than 0.08 and the Bentler–Bonett index or normed fit index greater than 0.90 [44]. In addition, discrepancies between the indexes (SRMR, unweighted least squares discrepancy (dULS), geodesic discrepancy (dG)) do not exceed the bootstrap-based percentile of 95% (HI95) or 99% (HI99), suggesting a good fit of the measurement model [45].

### 3.4. Measurement Model Assessment

As this is a training model, the measures do not need to be correlated, assuming that they are free of error, so the traditional assessment of validity and reliability is considered not to be applicable [46].

First, the collinearity between the indicators and the relevance and significance of the external weights were evaluated, showing that the collinearity indicators of the model were all values lower than 3.3 (1.03–2.21), as suggested by Diamantopoulos and Siguaw [47].

Table 5 and Table 6 show that there were weights that were not significant (EPC→CS; INT→CS; CUR→CS; REL→PNT; DSP→B), and so we checked the loads [48]. Although non-significant loads were observed in the CUR and EPC indicators of the controlling style construct, we decided to maintain the structure as postulated by Marin-Garcia and Alfalla-Luque [49], because the scale was already validated and the theoretical model was congruent.

### 3.5. Structural Model Assessment

Since the path coefficients were estimated on the basis of ordinal least squares (OLS) regressions, we needed to avoid the presence of multicollinearity. The results obtained do not show problems of collinearity, since the values of VIF are less than 5 [50].

Another aspect to evaluate in this section is the algebraic sign, magnitude and significance of the path coefficients (standardized regression coefficients) that show the estimates of the structural model relationships. As shown in Table 7, the sign of the hypotheses coincides with the sign of the coefficients. Furthermore, direct effects between variables and an indirect effect of 0.35 are presented; t (0.05;4999) = 5.645 between the controlling style and burnout latent variables.

The assessment of the determination coefficient (R2) indicates the amount of variance of a construct that is explained by the predictor variables of that construct in the model. The results show moderate power (psychological need thwarting: R2 = 0.44 and adjusted R2 = 0.43; burnout: R2 = 0.28 and adjusted R2 = 0.27), according to Chin [40].

Finally, in the structural model assessment, the effect size (f2) is evaluated [51], with controlling style showing a large effect with psychological need thwarting (0.39) and psychological need thwarting showing a large effect with burnout (0.77).

## 4. Discussion

The purpose of this research was to analyze the relationships between a controlling interpersonal style, psychological need thwarting and burnout in adolescent soccer players. In addition, a structural equation model was used to check whether (a) a controlling interpersonal style was a predictor of psychological need thwarting and whether (b) psychological need thwarting was a predictor of burnout. The results show relationships between the constructs studied and confirm the study hypotheses, supporting previous studies that have highlighted these associations. Specifically, the predictive capacity of a controlling interpersonal style for psychological need thwarting and of psychological need thwarting for burnout has been determined through the structural equation model.

The results obtained support the first hypothesis, since the perception of a controlling style acted as a positive predictor of basic psychological need thwarting. These results are related to those obtained in a longitudinal study with young soccer players by Balaguer et al. [16], where it was observed that changes in the perception of the coach’s autonomy support predicted changes in need satisfaction, and when the coach adopted authoritarian behavior, putting pressure on the athletes, they felt oppressed, incompetent and rejected. In another study by Castillo et al. [32], similar relationships were found between interpersonal control style and basic psychological need thwarting; in this study, however, each need was analyzed separately, and it was observed that a controlling style had significant predictive power for the thwarting of each of them (autonomy, competence and relatedness) and that these in turn acted as positive predictors of burnout.

The second hypothesis is confirmed by the results obtained in the study, which show that a perception of basic psychological need thwarting acts as a positive predictor of burnout. These results coincide with those of Bartholomew et al. [12]; in their study, a controlling style positively predicted the undermining of basic psychological needs and this in turn was positively related to burnout. Moreover, Balaguer et al. [16] observed that a controlling style was positively associated with basic psychological need thwarting, and this with burnout. Other studies in which basic psychological need thwarting was tested also support these results. The study by González, Castillo, García-Merita and Balaguer [52] confirms the importance of studying this issue as well as the finding that a controlling style is an antecedent to the ill-being of young athletes.

The results have shown that the most contributory indicators in the model are negative conditional support in controlling style, autonomy and competence needs in basic psychological need thwarting and a reduced sense of accomplishment in burnout. The data obtained in the correlation analysis and the structural equation analysis confirm the objectives set out and satisfy the claims of this study, confirming the conclusions reported by other authors who have highlighted these associations in other contexts [16,34], since the studied subscales of the controlling coaching style have been shown to predict basic psychological need thwarting, and these in turn have been shown to predict burnout. In our study, the weight of each indicator within the model was evaluated, providing more detailed knowledge of the nature of each variable as well as enabling more effective intervention in the areas that allow the appearance of a controlling style, basic psychological need thwarting and burnout.

We found that within the controlling style variable, the indicator with the most weight was negative conditional support, which could be defined as the withholding of attention and affection when the desired behaviors are not shown by the players [53]. As for the psychological need thwarting variable, the indicators that showed the most weight were autonomy and competence thwarting; this is in line with previous studies that have shown that autonomy thwarting is directly related to ill-being and that satisfaction of autonomy and competence contribute significantly to well-being [54]. As for the burnout variable, the indicator that carried the most weight was the players’ reduced sense of accomplishment with regard to their sports skills and abilities.

This study complements others that address the need to analyze coaching style in greater depth to gain a better understanding of which coaching behaviors cause athletes to be unable to enjoy their sports experience [5]. The results are similar to those of previous studies, although our research provides a series of methodological elements that contribute, in our view, to consolidating the findings. In this study, generalizability analyses were proposed and the structural equations model was applied using the partial least squares (PLS) method. This can be seen from a conceptual point of view, which has already been discussed, and from a structural perspective. Thus, although the results are similar to previous studies, the analyses we have performed increase their robustness. They confirm that these results are accurate and also generalizable, which is very important for extrapolating the findings to the population as a whole. Furthermore, although the structural equation model has already been proposed previously, the age range of the sample has been extended here, amplifying the findings of previous studies [5,32].

For athletes to perform their activity in an increasingly self-determined manner, coaches must develop an autonomy-supportive style that conveys the meaning of the activities they perform, supports freedom, promotes autonomy and involves athletes in decision-making processes [55]. Furthermore, it is not only at the adolescent stage that athletes need their coaches to support their autonomy; in older high-level competitive athletes, autonomy support by coaches also promotes feelings of autonomy and well-being.

This study has a series of limitations. First, other variables such as parental behavior, relationships with friends outside the sports context or self-perception of motor competence could affect the results. Second, the coaching time (at least one season) may be insufficient in some cases to establish a strong perception of interaction style. Although we considered it long enough for our purposes, other studies have used samples of athletes with a more long-standing relationship with their coaches of two or three years. It would therefore be interesting, in future studies, to analyze this question in order to determine what period of coexistence is necessary to adequately assess these parameters and what intrapersonal variables could determine it. As our results have shown, a controlling style is a positive predictor of basic psychological need thwarting and the latter is a predictor of burnout, and this information can be very useful for professionals who work with grassroots soccer players so that this relationship can be taken into account in the processes of training these athletes.

## 5. Conclusions

The results found in this study indicate that a controlling coaching style predicts basic psychological need thwarting in adolescent soccer players. In addition, the latter variable has been shown to be a predictor of burnout. As well as these direct effects, the results reveal an indirect relationship between controlling style and burnout.

## Figures and Tables

**Table 1:** Generalizability analysis.

Generalizability Analysis Variables	All Questionnaires	CCBS	PNTS	ABQ
	(q) (f)/(p)	(f)/(p)	(f)/(p)	(f)/(p)
Participants (p)	(103; INF)	(103; INF)	(103; INF)	(103; INF)
Questionnaire (q)	(3; INF)			
Factors (f)	(4; INF)	(4; INF)	(3; INF)	(3; INF)
Total observations	1236	412	309	309
Relative G coefficient	0.95	0.81	0.88	0.99
Absolute G coefficient	0.94	0.78	0.80	0.97
Relative error	0.01	0.01	0.01	0.01
Absolute error	0.01	0.01	0.02	0.01
SD (relative error)	0.09	0.11	0.10	0.05
SD (absolute error)	0.10	0.12	0.13	0.08

Note: CCBS: Controlling Coach Behaviors Scale; PNTS: Psychological Need Thwarting Scale; ABQ: Athlete Burnout Questionnaire; INF: infinity.

**Table 2:** Descriptive statistics.

Study Variables	M	SD	Skewness	Kurtosis	K-S
PEE	1.54	0.71	1.40	1.02	1.21
RSA	2.45	0.75	0.17	−0.26	0.65
DSP	1.88	0.91	1.11	0.53	1.35
AUT	2.91	1.32	1.05	1.62	1.18
REL	2.38	1.10	0.84	0.96	1.12
COM	2.75	1.44	0.58	−0.78	1.07
CUR	2.62	1.24	0.69	−0.23	1.01
NCS	2.56	1.16	0.75	0.53	0.91
INT	2.17	1.04	0.85	0.26	1.02
EPC	2.16	1.34	1.49	2.04	0.87

Note. PEE: physical/emotional exhaustion; RSA: reduced sense of achievement; DSP: devaluation of sports practice; AUT: autonomy; REL: relatedness; COM: competence; CUR: controlled use of rewards; NCS: negative conditional support; INT: intimidation; EPC: excessive personal control.

**Table 3:** Correlation level between Controlling Coach Behaviors Scale, Psychological Need Thwarting Scale and Athlete Burnout Questionnaire factors.

Study Variables	1	2	3	4	5	6	7	8	9
1. PEE	-								
2. RSA	0.45 **	-							
3. DSP	0.67 **	0.57 **	-						
4. AUT	0.45 **	0.49 **	0.54 ***	-					
5. REL	0.08	0.18	0.21 *	0.27 **	-				
6. COM	0.26 **	0.54 **	0.39 **	0.53 **	0.49 **	-			
7. CUR	0.06	0.12	0.03	0.13	0.14	0.15	-		
8. NCS	0.33 **	0.32 **	0.39 **	0.43 **	0.23 *	0.45 **	0.04	-	
9. INT	0.14	0.18	0.16	0.24 *	0.08	0.27 **	0.11	0.35 **	-
10. EPC	0.18	0.08	0.21 *	0.29 **	−0.15	−0.02	−0.07	0.28 **	0.34 **

Note: PEE: physical/emotional exhaustion; RSA: reduced sense of accomplishment; DSP: devaluation of sports practice; AUT: autonomy; REL: relatedness; COM: competence; CUR: controlled use of rewards; NCS: negative conditional support; INT: intimidation; EPC: excessive personal control. * *p* < 0.05; ** *p* < 0.01; *** *p* < 0.001

**Table 4:** Model fitting rates (estimated model).

Adjustment Measurement	Original Value		HI95		HI99	
	EM	SM	EM	SM	EM	SM
SRMR	0.06	0.05	0.07	0.07	0.08	0.07
dULS	0.18	0.16	0.25	0.23	0.32	0.30
dG	0.06	0.06	0.09	0.08	0.11	0.10
NFI	0.90	0.90				

Note. EM: estimated model; SM: saturated model; SRMR: standardized root mean square residual; dULS: unweighted least squares discrepancy; dG: geodesic discrepancy; NFI: normed fit index; HI95: 95% bootstrapping quantile; HI99: 99% bootstrapping quantile.

**Table 5:** Weights.

Study Variables	Original Sample	Sample Average	Standard Deviation	T-Statistics	*p*-Values
NCS→CS	0.84	0.79	0.13	6.36	0.000
EPC→CS	0.05	0.04	0.22	0.20	0.420
INT→CS	0.22	0.22	0.19	1.16	0.122
CUR→CS	0.24	0.22	0.17	1.36	0.087
AUT→PNT	0.61	0.60	0.17	3.49	0.000
COM→PNT	0.57	0.55	0.18	3.04	0.001
REL→PNT	−0.09	−0.08	0.11	0.75	0.225
RSA→B	0.61	0.60	0.16	3.62	0.000
DSP→B	0.26	0.24	0.18	1.39	0.081
PEE→B	0.31	0.31	0.15	1.97	0.024

Note. NCS: negative conditional support; EPC: excessive personal control; INT: intimidation; CUR: controlled use of rewards; AUT: autonomy; COM: competence; REL: relatedness; RSA: reduced sense of accomplishment; DSP: devaluation of sports practice; PEE: physical/emotional exhaustion; CS: controlling style; PNT: psychological need thwarting; B: burnout.

**Table 6:** Loads.

Study Variables	Original Sample	Sample Average	Standard Deviation	T-Statistics	*p*-Values
NCS→CS	0.94	0.88	0.07	12.16	0.000
EPC→CS	0.33	0.32	0.23	1.41	0.079
INT→CS	0.55	0.53	0.15	3.71	0.000
CUR→CS	0.29	0.26	0.18	1.54	0.062
AUT→PNT	0.89	0.87	0.07	11.43	0.000
COM→PNT	0.85	0.82	0.10	8.09	0.000
REL→PNT	0.36	0.34	0.13	2.58	0.005
RSA→B	0.90	0.88	0.07	12.65	0.000
PEE→B	0.75	0.74	0.09	7.63	0.000
DSP→B	0.82	0.80	0.09	8.70	0.000

NCS: negative conditional support; EPC: excessive personal control; INT: intimidation; CUR: controlled use of rewards; AUT: autonomy; COM: competence; REL: relatedness; RSA: reduced sense of accomplishment; PEE: physical/emotional exhaustion; DSP: devaluation of sports practice; CS: controlling style; PNT: psychological need thwarting; B: burnout.

**Table 7:** Path coefficients.

Study Variables	Original Sample	Standard Deviation	T-Statistics	*p*-Values
CS→PNT	0.53	0.07	7.85	0.000
PNT→B	0.66	0.06	11.91	0.000

Note. CS: controlling style; PNT: psychological need thwarting; B: burnout.
